# Are All Placebo Effects Equal? Placebo Pills, Sham Acupuncture, Cue Conditioning and Their Association

**DOI:** 10.1371/journal.pone.0067485

**Published:** 2013-07-31

**Authors:** Jian Kong, Rosa Spaeth, Amanda Cook, Irving Kirsch, Brian Claggett, Mark Vangel, Randy L. Gollub, Jordan W. Smoller, Ted J. Kaptchuk

**Affiliations:** 1 Psychiatry Department, Massachusetts General Hospital and Harvard Medical School, Charlestown, Massachusetts, United States of America; 2 Program in Placebo Studies, Beth Israel Deaconess Medical Center, Harvard Medical School, Boston, Massachusetts, United States of America; 3 School of Psychology, Plymouth University, Plymouth, United Kingdom; 4 Division of Cardiovascular Medicine, Harvard Medical School, Boston, Massachusetts, United States of America; 5 Biostatistics Department, Harvard School of Public Health, Boston, Massachusetts, United States of America; 6 MGH/MIT/HMS Athinoula A. Martinos Center for Biomedical Imaging, Charlestown, Massachusetts, United States of America; The James Cook University Hospital, United Kingdom

## Abstract

Placebo treatments and healing rituals have been used to treat pain throughout history. The present within-subject crossover study examines the variability in individual responses to placebo treatment with verbal suggestion and visual cue conditioning by investigating whether responses to different types of placebo treatment, as well as conditioning responses, correlate with one another. Secondarily, this study also examines whether responses to sham acupuncture correlate with responses to genuine acupuncture. Healthy subjects were recruited to participate in two sequential experiments. Experiment one is a five-session crossover study. In each session, subjects received one of four treatments: placebo pills (described as Tylenol), sham acupuncture, genuine acupuncture, or no treatment rest control condition. Before and after each treatment, paired with a verbal suggestion of positive effect, each subject's pain threshold, pain tolerance, and pain ratings to calibrated heat pain were measured. At least 14 days after completing experiment one, all subjects were invited to participate in experiment two, during which their analgesic responses to conditioned visual cues were tested. Forty-eight healthy subjects completed experiment one, and 45 completed experiment two. The results showed significantly different effects of genuine acupuncture, placebo pill and rest control on pain threshold. There was no significant association between placebo pills, sham acupuncture and cue conditioning effects, indicating that individuals may respond to unique healing rituals in different ways. This outcome suggests that placebo response may be a complex behavioral phenomenon that has properties that comprise a state, rather than a trait characteristic. This could explain the difficulty of detecting a signature for “placebo responders.” However, a significant association was found between the genuine and sham acupuncture treatments, implying that the non-specific effects of acupuncture may contribute to the analgesic effect observed in genuine acupuncture analgesia.

## Introduction

Placebo treatments and healing rituals have been used since the beginning of human history [1], [2]. The systematic study of placebo and ritual is still in its infancy [3], [4]. Whether all placebo treatments, or medical rituals, have equivalent effects remains unknown. This raises the question: Do patients who respond to one placebo intervention also tend to respond to other placebo interventions?

In a previous clinical trial [5] of chronic pain patients, we found that sham acupuncture reduced pain significantly more over time than did placebo pills, while placebo pills offered more short-term benefits of improving pain-disturbed sleep over sham acupuncture. Thus it showed that not all placebo treatments are equal. However, this clinical trial involved multiple, concurrent experimental arms and was not designed to answer the question of whether individuals who tend to respond to sham acupuncture also tend to respond to placebo pills.

In another study [6], Colloca and colleagues compared the placebo effects of verbal suggestion and conditioning to a control condition and found that verbal suggestion alone could not produce significant differences in subjective pain ratings. Conditioning, on the other hand, could significantly reduce subjective pain ratings. The between-subject design of this study prevents the authors from elucidating the association between verbal suggestion and conditioning effects.

Elucidating the relationship between different placebo modalities paired with verbal suggestion (suggestion-evoked placebo effects) as well as understanding their association with conditioning-evoked placebo effects will enhance our understanding of the variability observed in the placebo response. We are particularly interested in whether placebo responses can be characterized by the involvement of relatively stable *traits* or *states,* depending on particular circumstances.

Acupuncture has been used to relieve pain in East Asia for two thousand years [7]. Recent clinical trials investigating acupuncture's effects on chronic pain have shown contradictory results and often fail to show superiority over sham acupuncture [8]. This ambiguity may be the result of acupuncture's sizeable placebo effects as well as large inter-individual variability in response to acupuncture treatment [5], [8]–[11]. It is well known that some patients respond well to placebo and acupuncture treatments while others do not [5], [9]. Thus, as a secondary aim, this paper also addresses whether individuals who respond to sham acupuncture also respond to genuine acupuncture.

In this study, all subjects participated in two experiments sequentially. The first experiment was a multi-session crossover study [12] designed to test the analgesic effects of placebo Tylenol (pill), sham acupuncture, and genuine acupuncture (electroacupuncture) as compared to a no treatment (rest control). Subjects also participated in experiment two, in which we investigated how visual cues could modulate pain perception. These two experiments allow us to investigate the relationship between the analgesia evoked by different treatments as well as the analgesic effect evoked by conditioning cues.

## Methods

### Experiment one

#### Subjects

Seventy-one healthy, acupuncture-naïve subjects were recruited from the community, using flyers and postings, and enrolled in the study. The Institutional Review Board at Massachusetts General Hospital approved the study and all subjects gave written informed consent before commencing experiment one. All subjects were told that if they completed experiment one, they would also be invited to participate in experiment two. Subjects were debriefed at the end of experiment two and investigators explained the rationale for deception during the experiments. All subjects found study procedures acceptable and agreed to have their data used for analysis.

#### Outcome measurements

The outcome measures for this study include heat pain threshold and tolerance and subjective pain ratings of calibrated heat pain. All pain assessments were appliedusing a TSA-2001 Thermal Sensory Analyzer with a 3×3 cm probe (Medoc Advanced Medical Systems, RimatYishai, Israel) running proprietary computerized visual analog scale software (COVAS).

Subjects' thresholds and tolerances to pain were assessed on the dorsal portion of the hand using an ascending method of limits paradigm with a rate of rise of 0.5°C/sec from a baseline of 32°C. The 3 cm X 3 cm thermode was held lightly in place on the skin by a member of the study staff blinded to treatment mode.Subjects pressed a button in front of them to indicate when the heat “first becomes painful”to indicate pain threshold and when the heat “becomes too painful to tolerate” to indicate tolerance. Three trials of pain threshold and tolerance assessments were performed. The thermode was repositioned between each trial.

Calibrated heat pain stimuli were applied on the volar side of the forearms. All stimuli were delivered in 10-secondsegments (including an approximate 2.5 second ramp up and down from baseline) with a minimum inter-stimulus interval of 24 seconds. After each stimulus, the subjects were asked to rate the pain using the Gracely Sensory Scale (0–20) [13], [14].

#### Experimental procedure

**Figure 1:**
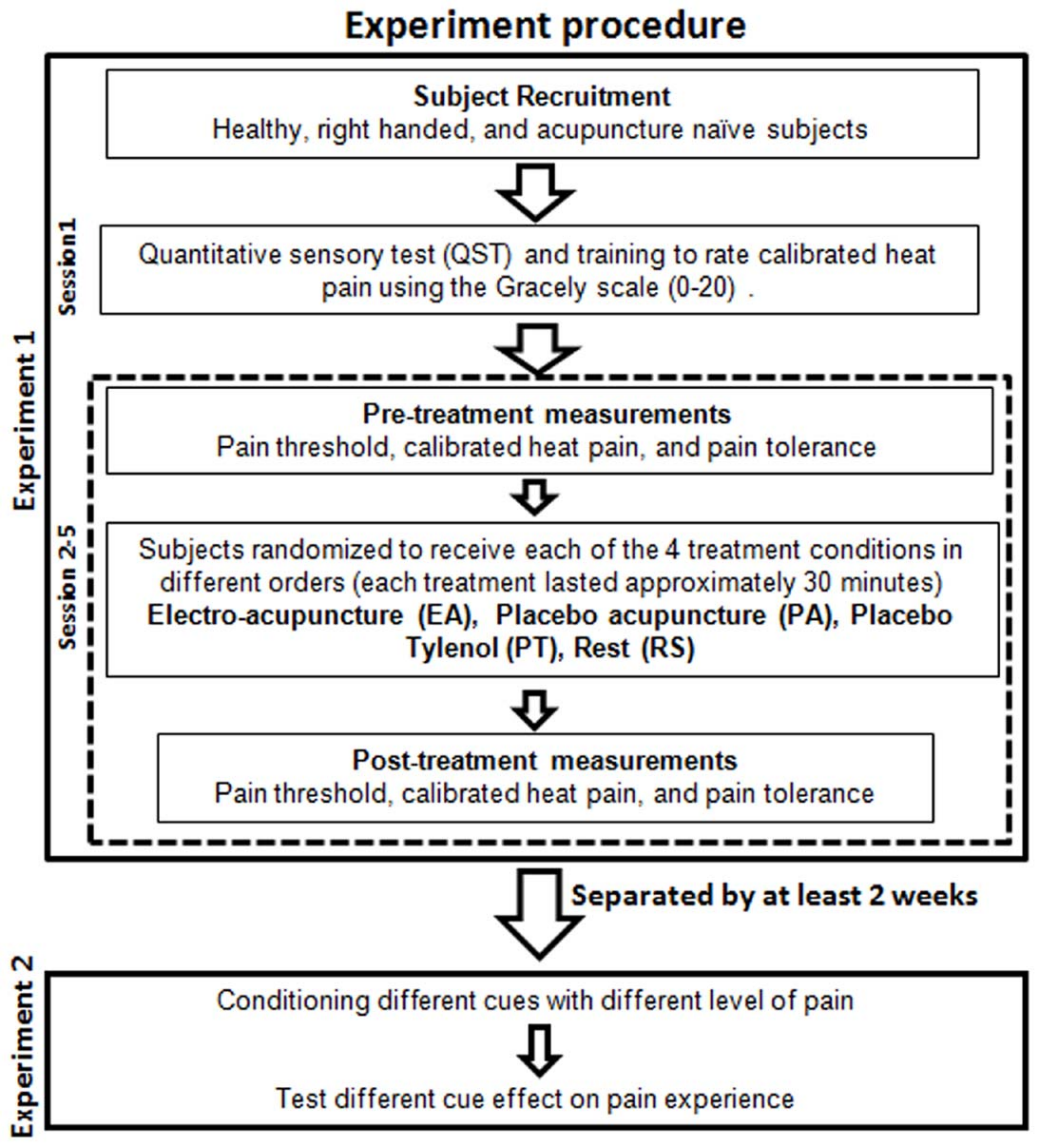
Experimental design.

The first experiment was a crossover study that involved five study visits, including one training session and4experimental sessions (**Figure** **1**). Each session was separated by at least 3 days to avoid sensitization to the repeated application of the noxious stimuli and to allow for full recovery of the subjects' skin. Similar methods have been used in our previous studies and no damage or lesions have been observed [12], [15]–[17].

The first session was used to introduce the study procedures, determine appropriate stimulus intensities for each subject, minimize anticipatory anxiety to pain and acupuncture, and to control for rating strategy and learning effects.

At the beginning of the training session (Session 1),subjects were told that in the subsequent 4 sessions, they would receive each of the 4 experimental conditions in a randomized order, i.e. two different modes of acupuncture treatment(electroacupuncture and manual acupuncture), one painkiller (Tylenol), and one control condition. Subjects were also told that before and after each treatment condition and the control condition, trained study staff would test their pain sensations to investigate the analgesic effect of different treatments on experimental pain applied to the forearm.

In reality, the manual acupuncture was sham acupuncture and the Tylenol painkiller was a placebo pill. In addition, subjects were explicitly informed that the control condition was a baseline control for the study and, thus, no treatment effects were expected. To maintain uniform expectancy across treatment sessions, subjects were also told that the effects of the two types of acupuncture (electroacupuncture and manual acupuncture) and Tylenol treatments could work via different mechanisms, and that the efficacy of one treatment would not influence the efficacy of the others.

The subjects were then trained to assess their pain threshold and tolerance. The heat stimuli were applied to the back of their hand in three separate locations for both threshold and tolerance measures. After that, they were instructed how to use the Gracely Sensory and Affective Scales [13], to rate calibrated heat pain with methods used in our previous studies [12], [15]–[17]. In order to identify the individually calibrated heat pain stimuli, a training set of ascending heat pain stimuli was administered starting from 38°C and increasing by one degree for each stimulus. From the subject's ratings of this initial series of stimuli, two heat pain intensities were determined for each subject: one to elicit responses in the strong range (“High pain”; 14–17 on the Sensory Scale) and one to elicit responses in the mild to moderate range (“Low pain”; 8–11 on the Sensory Scale). Sequences of the subject-specific High and Low stimuli were then applied in random order to separate areas of skin on the right volar forearm to further test the reliability of these ratings. Each random sequence consisted of six stimuli:3 Low pain stimuli and 3 High pain stimuli. The high and low temperatures were adjusted as necessary to reliably elicit a High or Low rating from a subject. Following these adjustments, the temperatures would remain fixed for all subsequent sessions.

Next, acupuncture was administered for 2 minutes to introduce subjects to the acupuncture experience. As several previous studies [18]–[20] have suggested that optimism is associated with placebo effects, all subjects were asked to complete the Life Orientation Test (LOT) to assess individual differences in generalized optimism versus pessimism.

Study sessions 2–5 were experimental test sessions. In each experimental session, a predetermined set of experimental heat pain tests was administered before and after treatment/conditioning in the following order: 1) pain threshold (3 times) on the dorsal portion of the right hand; 2) a pseudorandom noxious thermal stimuli sequence consisting of the 3 High and 3 Low pain stimuli on the volar side of right forearm; and 3) heat pain tolerance on the dorsal portion of the right hand. The dorsal portion of the hand was chosen due to its proximity to the acupuncture point used in this study. Heat pain was applied to separate areas of skin on the volar forearm in order to avoid the potential influence of one sequence of calibrated pain on each subsequent sequence. This predetermined set of tests was repeated during each experimental session. The only difference among the sessions was the treatment condition: electroacupuncture, sham acupuncture, placebo Tylenol, or rest control condition. The order in which subjects received the 4 experimental conditions was randomized prior to study proceedings using a computerized random number generator. Sequentially numbered method was applied for allocation concealment.

To ensure that all subjects could consistently rate the discrete levels of heat pain, only subjects who demonstrated the ability to distinguish different pain intensities during Session 2 (i.e., on average, rating the High pain stimuli higher than the Low pain stimuli after application of the random noxious stimuli sequence) were selected to proceed in the study.

Studies have shown that expectation can significantly influence an individual's perception of pain [15]–[17],[21]–[28]. Thus, all subjects were asked to complete the Expectations for Relief Scale (ERS) to indicate how much pain relief they expected from each particular treatment after receiving the treatment but prior to the post-treatment pain testing. The ERS is a ten-point scale (0–10) where 0 indicates a very negative expectation of “does not work at all” and 10 indicates a very positive expectation of “complete pain relief.”For the three treatment conditions, the subjects were told prior to treatment that they were about to receive a treatment that the investigators believed would have an analgesic effect on their entire body, including the arm. This served as verbal suggestion for the subjects in the treatment groups. For the control condition (30-minute rest period), subjects were still required to use ERS to rate how they expected their pain sensitivity would change after a 30-minute break, although they received no treatment.

#### Interventions

Placebo Tylenol Pill: Subjects were informed that the goal of the session was to test the analgesic effect of a non-opioid analgesia pill, acetaminophen (Tylenol by brand), on experimental pain. They were also told that the pill would begin to take effect approximately half an hour after administration and that previous research has suggested that Tylenol can produce a general analgesic effect on the whole body, including the experimental pain applied to the forearms. After orally ingesting the pill (in reality an inert placebo pill), subjects waited about half an hour prior to the beginning of the post-treatment pain assessment.

Sham (Placebo) Acupuncture: Sham acupuncture was performed using the validated Streitberger sham acupuncture device [12], . A small plastic ring was covered by an opaque, thin covering and then placed over2non-acupoints after the acupuncturist disinfected the area with isopropyl alcohol. A placebo needle, which was visually indistinguishable from genuine acupuncture needles, was inserted into the center of the ring and held in place by the tape. Because the Streitberger placebo needle has a blunt tip and a retractable shaft, the needle did not actually penetrate the skin; however the subjects felt a sensation similar to that of a pinprick or a scratch. After insertion, the needles were kept in place for 25 minutes.

Two sham acupoints (sham large Intestine 4 and 3 (LI 4 and LI 3) were chosen [32].Sham acupoint LI 4 is located on the dorsum of the right hand, between the 1st and 2nd metacarpal bones, approximately in the middle of the 1st metacarpal bone on the ulnar side. Sham acupoint LI 3 is about one half cm above the metacarpal bones. Both needles were rotated until the subject reported some level of sensation. Then, all needles were left alone for the remainder of the procedure (about 23 minutes) without further manipulation. The subject was told that these proceedings constituted the manual acupuncture treatment.

Genuine Acupuncture: The acupoints LI 4 and LI 3 on the right hand were used for the genuine acupuncture treatment. These points have well-documented analgesic effects in laboratory experiments [12], [33], [34]. After the acupuncturist located the acupoint and disinfected it with isopropyl alcohol, a small plastic ring was placed over the acupoint and secured with a thin strip of sterile plastic tape. This covering was used to maintain subject blinding to genuine and sham acupuncture conditions.

A small alligator-type conducting clamp was then attached to each needle to create a circuit using an electroacupuncture device (OMS Medical Supplies IC-1107). A current was passed through the electrode at a continuous frequency of 2 Hz. The intensity of the stimulation was gradually increased to the highest level subjects could tolerate without the sensation of sharp pain. The electroacupuncture was applied for approximately 23 minutes. Immediately following each acupuncture treatment (genuine and sham acupuncture treatment), subjects quantified the sensations they felt around the stimulated acupoint using the MGH Acupuncture Sensation Scale (MASS) [12], [35].

No treatment rest control: The subjects in this group were told that the no-treatment condition was used as a within-subject control for the treatment conditions. Following the initial pain assessments, subjects were told to simply sit and relax for 30 minutes and wait for the post-treatment pain assessment to begin. Again, the subjects were asked to rate how much they thought the half hour rest period would change their sensitivity to pain during the second set of pain assessments.

### Experiment two

All subjects who completed experiment one were invited to return to our lab at least 2 weeks after completion of experiment one to participate a one-session fMRI study to investigate the conditioning effects of visual cues. Please see original publication for more details on experimental procedures and fMRI results [36]. The present manuscript only focuses on the analysis of the association between the placebo effects evoked by suggestion in experiment one and the visual cue conditioning effects in experiment two. This analysis has not been included in previous reports.

In brief, at the beginning of the experiment, subjects were told that the aim of experiment two was to investigate the brain's response to different levels of thermal pain. Subjects were then familiarized with the visual presentation paradigm, including a pre-stimulus cue, a pain stimulus symbol, and a post-stimulus rating scale. In addition, subjects were told that the pre-stimulus cue (text saying either “HIGH” or “LOW”) would indicate the level of the subsequent pain stimulus.

Based on experiment one, temperatures that elicited subjective intensity ratings in the strong range (“High pain”; 14–17 on the Sensory Scale) and one to elicit responses in the mild to moderate range (“Low pain”;8–11 on the Sensory Scale)were selected for each subject and used in experiment 2 (MRI study). Immediately prior to the fMRI scan, a brief pain sensitivity test was performed to further confirm that the subjective ratings in response to the high and low temperature stimuli elicited were within the targeted range for the study and necessary adjustments were made.

During fMRI scanning, 3 different series of pseudo-randomized pain sequences were applied to the distal portion of the right forearm above the wrist. Subjects were instructed to focus on a small black fixation cross in the center of the screen in front of them. The first scan was a contextual conditioning/learning scan where subjects were presented with a pre-stimulus cue, indicating (without deception) whether they would be administered a LOW or HIGH pain stimulus. The duration of the pain stimulus was 12 seconds and the intensity of the stimulus for this first sequence always corresponded to the pre-stimulus cue. After each pain stimulus, the Sensory Box Scale was displayed on the screen for 8 seconds, and subjects rated the intensity of their subjective pain by moving a cursor along the scale. In total, this learning sequence included 4 LOW and 4 HIGH pain stimuli.

The initial conditioning scan was followed by 2 test pain sequences in which the LOW cue (LC) was sometimes followed by the HIGH pain stimulus (HP) (the LC condition), representing a condition where subjects were expected to report less pain in response to a suggested low stimulus, and sometimes followed by the LOW pain stimulus. Both test scans included nine stimuli, where 3 of the stimuli were cued as HIGH pain and six were cued as LOW pain. Following all HIGH cues (HC), a high pain stimulus was delivered (the HC condition). However, following 3 of the six LOW cues, a HIGH pain stimulus was delivered (the LC condition) instead of a LOW pain stimulus. All other timing aspects of the 2 test scans were identical to the first contextual learning/conditioning scan. The subjective pain ratings evoked by the different cues (LC or HC with identical HIGH heat pain stimuli) in runs 2 and 3 were used to calculate the conditioned cue effect. The cue effect was used to investigate the association between the analgesic effect of treatment observed in experiment one and the analgesic effect of visual cue conditioning.

## Analysis

### Experiment one

The primary outcomes for experiment one were post-treatment measures of pain threshold, pain tolerance, and pain ratings (for high pain and low pain). Because each subject was evaluated under all 4 of the experimental conditions, the data were analyzed using repeated measures analysis of covariance (ANCOVA) with the corresponding baseline measures as the covariate and treatment as the factor of interest. A separate ANCOVA model was fit for each of the 3outcomes. In these ANCOVA models, session order and subject ID were also included as factors, eliminating the need to further control for subject-level covariates such as age and gender.

For each subject outcome, the distribution of residuals from the ANCOVA model was visually inspected. In the event that noticeable non-normality was detected, a robust analysis rank ANCOVA was performed, replacing both outcomes and baseline scores with their respective ranked values [37]. If both normal-theory and robust analyses produced similar results, we concluded that the results were not likely to be sensitive to the normality assumption.

For outcomes in which a significant difference among treatments was detected, regression analyses were used to investigate whether subject-level outcomes (including subject response to other treatments) were useful predictors of response to genuine acupuncture. Robust regression using M-estimation was employed to minimize the effect of outliers [38].All analyses were conducted using Stata (version 11). P-values of ≤0.05 were considered to be statistically significant.

### Experiment two

The primary outcome for experiment two was the subjective pain ratings evoked by the different visual cues (Low Cue or High Cue with identical HIGH heat pain stimuli) in the 2 test pain sequences. We explored the association between the analgesic effect evoked by different treatments in experiment one (genuine acupuncture, sham acupuncture and placebo pills compared with rest condition, separately) and visual cue effects in experiment two by applying non-parametric Spearman correlations separately. For this analysis, we used the primary outcomes of experiment one (changes in pain threshold, pain ratings of calibrated pain stimuli and pain tolerance), and the primary outcome in experiment two (conditioning cue effects as indicated by subjective pain rating changes to identical pain stimuli).

## Results

### Experiment one

Of the 71 healthy subjects who participated the study, 48 subjects (19 males, 34 white, 4 black, 6 Asian, 3 more than one race and 1 unknown) ages 21–37 (mean: 26.23, SE: 0.48) completed experiment one with data for analysis. One subject who completed experiment one was not included in data analysis due to missing data. Twenty-two subjects did not complete the study. The reasons for dropped subjects included scheduling difficulties (11), unstable pain ratings (6), inability to tolerate testing conditions (acupuncture or heat) (3), inability to understand nuances of pain rating scales (1), and voluntary withdrawal (1). The average intervals between treatments (baseline and each of the four conditions) were 8 days, 7.7 days, 7.5 days and 8.2 days.

**Figure 2:**
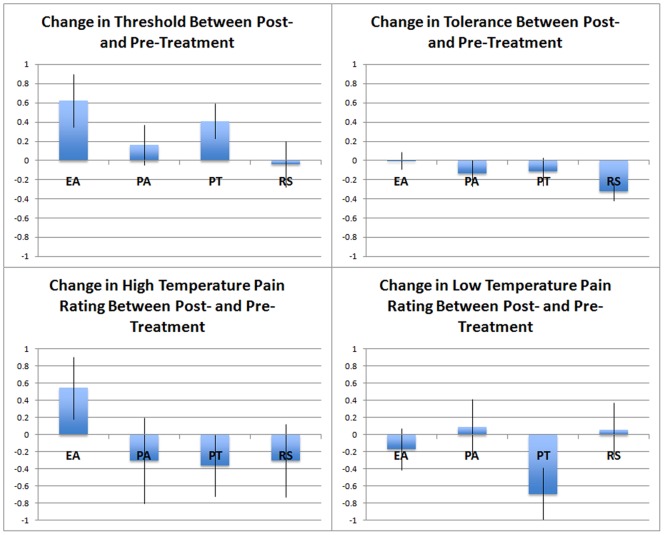
Summary of pain measurement difference (post- minus pre-treatment, mean ± SE) on pain threshold, pain tolerance, and pain rating across different groups. EA, electroacupuncture group; PA, placebo acupuncture group; PT, placebo Tylenol group; RS, resting control group.

A summary of pain threshold and tolerance values and pain ratings of calibrated heat pain stimuli are shown in Table 1 and Figure 2. The ANCOVA (Table 2) showed that pain threshold post-treatment scores were significantly dependent on the mode of treatment (F = 3.57; df = 3, 137; p = 0.016). The distribution of the post-treatment scores, as well as the residual values from the ANCOVA model, was found to include outliers, which could potentially impact the results of the ANCOVA model. However, the sensitivity analysis conducted using rank data found a similar result (F = 3.63; df = 3, 137; p = 0.015). Post hoc analysis among the 4 experimental conditions showed that both genuine acupuncture and placebo pills produced significant post-treatment pain threshold increases (+0.79, 95% CI:[+0.25, +1.33], p = 0.004; and +0.74,95%CI: [+0.19, +0.1.29], p = 0.008 respectively) relative to rest control. No other significant differences between treatments were detected with respect to pain threshold (Table 3).

**Table 1:** Pre- and post-treatment pain threshold (centigrade), tolerance (centigrade), and pain ratings (intensity rating of high and low pain stimuli using the 0–20 GracelyScale) across different conditions (mean±SE).

	Pain Threshold		Pain Tolerance		High Pain Rating		Low Pain Rating	
	Pre	Post	Pre	Post	Pre	Post	Pre	Post
Electro-acupuncture	42.2±0.5	42.9±0.5	48.2±0.3	48.2±0.2	12.1±0.4	12.6±0.4	4.6±0.4	4.4±0.4
Sham acupuncture	42.3±0.5	42.5±0.5	48.2±0.3	48.1±0.3	12.5±0.5	12.2±0.6	4.7±0.4	4.8±0.4
Placebo Pill	42.5±0.5	42.9±0.5	48.3±0.3	48.3±0.2	12.2±0.4	11.8±0.5	5.0±0.4	4.3±0.4
Rest Control	42.0±0.5	42.0±0.5	48.2±0.3	47.9±0.2	12.8±0.4	12.4±0.5	4.8±0.4	4.7±0.4

**Table 2:** Treatment effects across different measurements of pain sensation.

Outcome	Original Data (Y_ij_)		Rank Data (R_Yij_)	
	F-Statistic	p-value	F-Statistic	p-value
Pain Threshold	3.57 (df = 3,137)	0.016	3.63 (df = 3,137)	0.015
Pain Tolerance	2.35 (df = 3,134)	0.076	2.61 (df = 3,134)	0.054
Low Pain Rating	1.38 (df = 3,137)	0.251	0.77 (df = 3,137)	0.513
High Pain Rating	1.19 (df = 3,137)	0.317	0.83 (df = 3,137)	0.480

**Table 3:** Post-hoc analysis of pain threshold: effect of treatment relative to rest control.

Treatment	Effect	SE	p-value
Rest	(ref)	–	–
Placebo Tylenol	+0.74	0.28	0.008
Sham acupuncture	+0.39	0.27	0.153
Electroacupuncture	+0.79	0.27	0.004

For pain ratings of calibrated heat pain stimulation and pain tolerance, no significant treatment effects were detected (Table 2). While the null hypothesis of global F-test for the effect of treatment on the pain tolerance outcome could not be rejected, it should be noted that an exploratory post-hoc analysis using a test for detecting trends across the treatment groups did reveal a significant progression in the strength of treatment effects when the treatments were ordered as rest control condition<placebo Tylenol = sham acupuncture <electroacupuncture. In this analysis, the effect of treatment was estimated, with the rest condition coded as 0, each of the two placebo treatments coded as 1, and the genuine acupuncture coded as 2. Using the same ANCOVA models as mentioned above, the effects of treatment were found to be significant with p-values of 0.012 and 0.007 corresponding to the original and ranked data, respectively.

Because only the pain threshold outcome showed significant differences among the experimental conditions, further analyses focused only on this outcome. In an attempt to determine whether responses to placebo Tylenol were significantly related to each subject's response to sham acupuncture, we investigated the relationship between post-treatment placebo Tylenol pain threshold and the following predictors, controlling for pre-treatment placebo Tylenol pain threshold: pre- and post-sham acupuncture pain threshold change, pre- and post-electroacupuncture pain threshold change, pre- and post- rest control pain threshold change, expectancy ratings for each of the4 conditions(placebo Tylenol, sham acupuncture, genuine acupuncture, and rest control), age, gender, and optimism measured by the LOT scale.

We assessed these relationships via univariate and multivariate regression models. The results (Table 4) show the estimated regression coefficients and associated p-values. In the left column, each variable is considered one at a time, controlling only for baseline placebo Tylenol pain threshold and the session number in which placebo Tylenol was received. In the right column, all variables appear together in a single multivariate model. In both settings, there is no significant association between post-treatment placebo Tylenol pain threshold and the sham acupuncture pain threshold change, suggesting the 2 placebo modalities are not associated. No other predictors were found to be significantly related to placebo Tylenol pain threshold in either univariate or multivariate models.

**Table 4:** Potential predictors of placebo Tylenol response using pain threshold as outcome.

	Univariate Model		Multivariate Model	
Variable	Coef.	P-val	Coef.	P-val
Sham acupuncture Score Change	0.13	0.34	0.15	0.40
Electroacupuncture Score Change	0.03	0.80	0.05	0.69
Rest Score Change	−0.13	0.28	−0.13	0.45
Electroacupuncture Expectancy	−0.09	0.40	−0.01	0.95
Sham acupuncture Expectancy	0.00	0.99	0.00	0.98
Placebo Tylenol Expectancy	0.05	0.63	0.12	0.38
Rest Expectancy	−0.12	0.31	−0.16	0.36
Age	0.03	0.67	0.05	0.62
Male	0.21	0.64	0.30	0.63
Optimism	−0.01	0.90	0.01	0.89

Similarly, in an attempt to determine whether any individual characteristics were significantly related to subject response to electroacupuncture, we investigated the relationship between post-treatment electroacupuncture pain threshold and the following predictors, controlling for pre-treatment electroacupuncture pain threshold as well as the session number in which electroacupuncture was received: pre- and post- sham acupuncture pain threshold change, pre- and post-placebo Tylenol pain threshold change, pre- and post- rest control pain threshold change, expectancy ratings for each of the 4 conditions(genuine acupuncture, sham acupuncture, placebo Tylenol, and rest control), age, gender, and optimism measures using the LOT scale.

The results (Table 5) show the estimated regression coefficients and associated p-values. In both settings, the sham acupuncture pain threshold change was significantly and positively correlated to post- electroacupuncture pain threshold (β = 0.41, p = 0.005 in univariate model; β = 0.41, p = 0.03 in multivariate model).

**Table 5:** Potential predictors of electro-acupuncture response using pain threshold as outcome.

	Univariate Model		Multivariate Model	
Variable	Coef.	P-val	Coef.	P-val
Sham acupuncture Score Change	0.41	0.005	0.41	0.03
Placebo Tylenol Score Change	0.22	0.26	0.21	0.34
Rest Score Change	0.15	0.30	0.10	0.57
Electroacupuncture Expectancy	0.02	0.85	0.08	0.65
Sham acupuncture Expectancy	−0.06	0.54	−0.11	0.48
Placebo Tylenol Expectancy	0.03	0.75	−0.05	0.74
Rest Expectancy	0.10	0.48	0.10	0.61
Age	0.07	0.35	0.05	0.59
Male	0.00	0.99	0.04	0.96
Optimism	−0.04	0.51	−0.02	0.72

After each treatment, the expectancy for that particular treatment was also measured using the ERS. The average expectancy ratings (avg ± SE) for each of the 4 experimental conditions are: 4.4±0.3 for the genuine acupuncture condition, 4.1±0.4 for the sham acupuncture condition, 5.3±0.3 for the placebo Tylenol condition, and 0.6±0.2 for the rest control condition. The ANOVA showed that there was a significant difference in expectancy ratings across the 4 experimental conditions (F(3,137) =  79.6, p<0.0001), and post hoc analysis showed that expectancy rating in all 3 treatment groups were significantly greater than the rest control condition (p<0.001 for each). Expectancy ratings in the placebo Tylenol condition were also greater than both the sham acupuncture condition (p = 0.001) and the genuine acupuncture condition (p = 0.003).

To further test the association between the pre- and post-treatment pain measurement differences and expectancy ratings, we regressed all post-treatment pain measurements on expectancy scores, controlling for pre-treatment measurements, session order, and subject ID. The results indicated that the expectancy level was significantly correlated with pain threshold (beta  = 0.17, p<0.001) and pain tolerance (beta  = 0.07, p = 0.003), but not significantly associated with low and high pain ratings.

We also measured the sensations evoked by electro-acupuncture and sham acupuncture treatment using the MASS. A summary of the results is shown in Table 6. There was a significant difference in the average MASS sensation between electro-acupuncture (2.2±1.5) and sham acupuncture (0.5 ±0.6) conditions (p<0.001). Correlation analysis did not find significant correlations between the MASS rating and outcome measurements (pain threshold, pain tolerance and pain rating changes).

**Table 6:** Average MASS scores (mean ± SE) across electro-acupuncture and sham acupuncture conditions.

Acu Mode	Sore-ness	Aching	Deep Pressure	Heavi-ness	Fullness/ Distention	Tingling	Numb-ness	Sharp Pain	Dull Pain	Warmth	Cold	Throbbing
Verum	2.8±2.4	2.7±2.5	2.9±2.6	2.1±2.4	1.4±2.1	3.1±2.6	2.1±2.7	0.7±0.9	3.3±2.6	1.1±2.1	0.6±1.5	3.2±2.9
Sham	0.4±0.7	0.3±0.7	0.3±0.6	0.7±1.7	0.3±0.8	1.4±1.9	0.8±1.9	0.0±0.1	0.4±0.9	0.6±1.5	0.1±0.5	0.1 ±0.5

### Experiment two

Of the 48 datasets that were included in analyses from experiment one, data from 46 subjects were included in data analysis for experiment two. One subject was not able to complete experiment due to scheduling conflicts, and the other subject did not complete experiment two (fMRI scan session) due to a suspected abnormal brain scan. Subsequent follow up with a physician determined that the abnormality was not clinically significant.

Behavioral data of experiment two showed that the pain ratings (mean± SE) for the low-cue low pain condition averaged 5.3±0.3, the low-cue high pain condition (LC) averaged 11.3±0.4, and the high-cue high pain condition (HC) averaged 14.2±0.3.The cue effect (LC condition minus HC condition) ranged from −0.5 to 7.6 (median  = 3.0). A one-way mixed model was applied with mean pain ratings as the response, the result showed that the contrasts HC and LC condition was highly significant (p<0.0001).

To explore the association between the analgesic effects observed from experiment one and the conditioning cue effects observed in experiment two, we applied a Spearman correlation between the analgesic effect of different treatments (treatment (pre-post) – control (pre-post) and cue effects respectively. The results showed that that there was no association between the conditioning cue effect and any treatment-evoked analgesic effect (p-values ranged from 0.13–0.99).

## Discussion

In this study, we investigated the analgesic effects produced by placebo Tylenol, sham acupuncture, genuine acupuncture and a rest control condition, as well as the association between the effects of verbal suggestion on evoked placebo treatments (placebo Tylenol and sham acupuncture), electroacupuncture, and conditioning cue effects. The results showed that genuine acupuncture and placebo pills could significantly increase subjects' pain threshold compared with rest control condition. Regression analysis showed that there were no significant associations between individuals' responses to placebo pills, sham acupuncture, electroacupuncture and conditioning cue effects; however, subjects' responses to sham acupuncture correlated significantly with their response to genuine acupuncture.

To the best of our knowledge, the question of whether individuals are likely to respond to different placebo routes of administration in a similar or different manner was an explicit topic of research in several experiments performed between 1956 and 1965 and each concluded that sham injections were more powerful than placebo pills [39]–[42].Further supporting this belief, a relatively old meta-analysis that included 35 RCTs for treatment of acute migraine extracted the placebo responses of trials that utilized an oral placebo and compared those responses with placebo responses in trials that used an injected placebo. The analysis showed that the injected placebo was statistically and clinically superior to the oral placebo [43].

Our team performed an RCT comparing placebo pills and sham acupuncture across 270 patients with chronic arm pain. The results showed that sham acupuncture was statistically and clinically superior to placebo pills over time, while placebo pills were more beneficial in the short-term for improving sleep disturbances due to the pain condition [5]. A recent Cochrane meta-analysis of placebo effects [44] revisited this question of pill placebos versus device placebos and found that RCTs (n = 61) with “physical placebos” (which included sham acupuncture and such devices as sham electrotherapy and ultrasound) produced larger placebo effects than studies with “pharmacological” pill controls. In a separate study, Linde and colleagues [10] re-analyzed this meta-analysis [44] to investigate whether effects associated with sham acupuncture differed from those of other 'physical placebos'. They found pooled standardized mean differences were −0.41 (95% CI(−0.56, −0.24)) between sham acupuncture and no treatment, and −0.26 (95% CI −0.37, −0.15) between other physical placebos and no treatment, implying a larger effect of acupuncture treatment. In another meta-analysis from the same group [8], the authors also found that sham acupuncture interventions are often associated with moderately large nonspecific effects.

In our present study, we did not find any differences between the sham acupuncture treatment and placebo pills in the measurement of pain threshold, tolerance, and pain ratings. Additionally, there was no significant association between any of the pain measurement changes after the 2 modes of placebo treatments (placebo pill and sham acupuncture) in both univariate analysis and after adjusting for expectancy and optimism, suggesting a lack of consistency in placebo response across different modes of placebo treatments. This result is consistent with the findings from a recent study, in which Whalley and colleagues [45] found that placebo effects across trials were highly correlated when placebo creams bore the same name but were not significantly correlated when placebo creams had different names.

We did not find any significant sham acupuncture effects compared to the rest control. Additionally, analgesic effects across all treatment modes were, in general, mild. This is consistent with previous findings that indicate that placebo effects evoked by verbal suggestion alone are weak in healthy subjects compared to patients [6], [46], [47], particularly considering that acupuncture treatment is a novel treatment method in the United States [15].The fact that sham acupuncture had significantly lower expectancy ratings than placebo Tylenol further supports this view.

Interestingly, we found that response to genuine acupuncture is significantly and positively correlated with response to sham acupuncture. This finding suggests that a non-specific effect of acupuncture treatment may play a significant role in the analgesic effect evoked by genuine acupuncture, which is consistent with the conclusion from a recent meta analysis on acupuncture treatment of chronic pain [11]. Nevertheless, our results do not indicate that the two are necessarily the same. We found that although the explicit conscious expectancy for genuine acupuncture is lower than the expectancy for placebo pills, the analgesic effect (as measured by pain threshold and tolerance) produced by genuine electroacupuncture trends is higher than the effect produced by placebo pills. This dissociation suggests that the specific effect of acupuncture may significantly contribute to the analgesic effect of electroacupuncture, which is consistent with previous neuroimaging studies that found different brain activation patterns associated with placebo and acupuncture analgesia [16], [17].

In a previous study [6], Colloca and colleagues investigated the effects of both expectation, which was induced by verbal suggestion alone, and conditioning at the level of N1 and N2–P2 components of CO2 laser-evoked potentials (LEPs) and subjective pain reports. They found that both verbal suggestions (placebo cream) and conditioning modified the N2–P2 complex. Verbal suggestion in combination with the application of placebo cream could not produce subjective pain rating differences as compared with control condition, while conditioning with placebo cream produced more robust reductions of LEP amplitudes and subjective pain rating decreases. Our results are consistent with their findings of weak placebo effects with verbal suggestion alone and robust effects using a conditioning paradigm. Although we cannot perform a direct comparison, in our results the p-value of the conditioned visual cue effect was more significant compared to the placebo effect observed with verbal suggestion alone. More importantly, our results extend their findings showing that there is no association between the placebo analgesia observed with verbal suggestion and the effects of visual cue conditioning, suggesting that each may be associated with different mechanisms.

In this study, the most significant findings were pain threshold changes, but not pain tolerance or pain rating changes. Previous studies have suggested that different pain sensation measurements (pain threshold, pain tolerance, and pain rating) may represent different aspects of pain sensation [48], [49]. One potential explanation is the order effect of the pain measurements obtained. In this study, we collected pain threshold, then pain rating to calibrated heat pain, and finally the pain tolerance measurements. Since pain threshold is always measured first, this may partly explain why the greatest analgesia effect is observed in the pain threshold measurement. However, further study is warranted.

Although our study is based on an experimental model in healthy volunteers, it may have implications for clinical care and research. Until now it has been understoodthat aggregate placebo responses in RCTs and experiments are variable [50]. Our study may suggest a new dimension of variability in placebo effects: responses may differ within individual subjects according to route of administration (pills vs. sham acupuncture) as well as environmental cues and learning processes (e.g., verbal suggestion and conditioning). This finding implies that placebo responses may not be dependent on stable individual *traits* but rather are more a characteristic of the *state* circumstances of individuals or combination of both *trait* and *state*. This result may help explain the difficulty of detecting reliable and consistent placebo responders [51]. Furthermore, the fact that people have unique responses to different routes of administration suggests that, for some, pills may work better than injections or vice versa. Moreover, the significant correlation between genuine and sham acupuncture on subjective outcomes suggests that elucidating whether acupuncture is more than a placebo effect will be difficult.

The potential limitations of the study include the following: 1) the crossover design may have resulted in carryover effects and order effects from one study visit to the next. However, since the treatment sessions were separated by at least 3 days, we did not expect any residual effect of the previous treatment on the subsequent session. Additionally, each treatment session included pre-treatment measurements, which were used as covariates in our analysis. This should adjust for any residual effect from a previous treatment as well as other potential confounding factors, such as hyperalgesia due to repeated pain application; 2) The placebo effect of sham acupuncture was not statistically superior to no-treatment control. It is possible that had we had a larger placebo effect, we might have found a correlation between pill and sham device; 3) The study was performed on healthy subjects in a relatively small sample size and thus it is unclear whether or not similar results could be repeated in patient populations. These results warrant further investigation of this topic.

In summary, in this crossover study, we found that individuals respond differently to different types of verbally suggested placebo treatments and cue conditioning. In addition, we found a significant association between genuine and sham acupuncture treatments, which implies that non-specific effects may significantly contribute to the analgesic effect observed in acupuncture analgesia.
